# BOLD fMRI in the White Matter as a Marker of Aging and Small Vessel Disease

**DOI:** 10.1371/journal.pone.0067652

**Published:** 2013-07-02

**Authors:** Ilia Makedonov, Sandra E. Black, Bradley J. MacIntosh

**Affiliations:** 1 Heart and Stroke Foundation Centre for Stroke Recovery, Sunnybrook Health Sciences Centre, Toronto, Ontario, Canada; 2 Institute of Biomaterials and Biomedical Engineering, University of Toronto, Toronto, Ontario, Canada; 3 Department of Medicine (Neurology), Sunnybrook Health Sciences Centre and University of Toronto, Toronto Ontario, Canada; 4 Brain Sciences Research Program, Sunnybrook Research Institute, Toronto, Ontario, Canada; 5 Department of Medical Biophysics, University of Toronto, Toronto, Ontario, Canada; University of California, San Francisco, United States of America

## Abstract

**Purpose:**

Determine whether white matter signal fluctuation on T2* weighted BOLD contrast images are associated with aging and cerebral small vessel disease (SVD).

**Methodology:**

Resting state BOLD data were collected with a 250 ms repetition time (TR) to achieve unaliased, ungated cardiac sampled BOLD (cs-BOLD) images on 11 young adult controls, 10 healthy older adult controls and 7 adults with extensive white matter hyperintensities (WMH) from SVD. Tissue classes (WM and GM) were segmented on T1 images. WMH were identified on FLAIR images in the SVD group. Raw physiological noise (σ_physio_) and cardiac pulsatility (i.e. fluctuations at the cardiac frequency) were calculated voxel wise and group differences were tested by ANOVA. It was also possible to calculate σ_physio_ in 2s TR cardiac aliased whole-brain BOLD (wb-BOLD) data (N = 84) obtained from the International Consortium for Brain Mapping.

**Results:**

CS-BOLD metrics showed an aging and SVD effects (p<0.0005). Covariates such as thermal noise, WM volume and partial volume did not influence the significant aging effect seen on the cardiac pulsatility metric (p<0.017) but did influence the σ_physio_ (p = 0.184). As a verification of the cs-BOLD findings, the wb-BOLD also showed a linear aging effect of σ_physio_ in WM. In the SVD adults, cardiac pulsatility and σ_physio_ were lower in WMH regions compared to normal appearing white matter (NAWM) regions (p<0.0013 and p<0.002, respectively). Cardiac pulsatility was better able to distinguish WMH regions from NAWM than σ_physio_ as measured by effect size (Cohen’s d 2.2 and 0.88, respectively).

**Conclusion:**

NAWM was found to have graded increases in cardiac pulsations due to age and SVD, independently. Within SVD participants, WMH lesions had reduced physiological noise compared to NAWM. Cardiac pulsatility in resting BOLD data may provide a complementary dynamic measure of WM integrity to add to static FLAIR anatomical images.

## Introduction

BOLD-contrast functional MRI (fMRI) time series data are influenced by physiological sources such as respiration, pulsatile blood flow, vasomotion, spontaneous fluctuation in cerebral metabolism (CMRO_2_), and variation in respiratory and cardiac rates [1]. These signals may be regarded as undesirable features in fMRI data and are hence referred to as physiological noise. Numerous studies have focused on isolating these physiological components [2]–[6] since it was established early on that these features seem to vary between populations. The noise component in cortical grey matter (GM) is known to increase with age, for example [7]. The purpose of this study is to extend this line of research by characterizing physiological noise within white matter (WM) in young adults, older healthy adults and older adults with white matter disease.

White matter hyperintensities are often referred to as leukoaraiosis and are associated with numerous issues related to executive function, processing speed, and immediate and delayed memory [8]; motor and gait disturbances [9]; urinary dysfunction [10]; and mood disorders [11]–[12]. WMH is also a predictor of stroke, dementia, and death [13]. Volumetric WMH lesion burden predicts cognitive decline in normal elderly adults and progression to dementia in adults with amnestic mild cognitive impairment [14].

Questions remain about the cerebrovascular risks that may accompany WMH lesions. For example, it not clear how to interpret the total WMH lesion volume as a risk factor since it appears that the lesion volume is only weakly correlated with cognitive performance in previous cohort [15] and community populations [16] studies. Furthermore, mean diffusivity using diffusion tensor imaging is correlated with executive function and IQ more strongly than WMH lesion volume [17]. To advance our understanding of the sequaele of WMH, there is a need to identify new ways to characterize WM risk and burden.

WMH are thought to be an indication of cerebral small vessel disease (SVD) [18], therefore hemodynamic measures have helped to advanced the body of knowledge. Cerebral blood flow (CBF) is reduced within WMH regions, as assessed by perfusion-weighted MRI [19], arterial spin labelling [20], and single photon emission computed tomography (SPECT) [21]. Cerebrovascular reactivity also appears to be reduced in WMH regions [19]. These observations provide evidence that implicates hemodynamic processes. On the arterial side, increased stiffness will cause higher pulsatile energy transmission further down the vascular network towards smaller vessels in aging [22] and in WMH cohorts [23]. On the other side of the circulation in WM, occlusive venous collagenosis has been reported in WMH and this phenomenon could also contribute to upstream mechanical damage [24]–[25].

In the current study we investigate whether global indices of WM BOLD physiological noise can be used to characterize WM as a function of aging and in the presence of SVD. Two candidate physiological noise metrics are introduced: 1) physiological noise across the entire temporal frequency spectrum and 2) the amplitude of physiological noise specifically at the principal cardiac frequency. The former metric can be used on high or low temporal resolution BOLD data while the latter requires cardiac unaliased sampling and is expected to be more specific to cardiac effects in the time series. It is hypothesized that both physiological noise metrics within the normal appearing white matter (NAWM) will be: 1) larger in healthy elderly controls compared to healthy young controls and 2) larger in SVD patients compared to age matched elderly controls. Within the SVD cohort, a direct comparison between WMH and NAWM regions was also performed. As a post-hoc analysis, publically available resting-state BOLD data were accessed and an aging effect in WM voxels was performed to verify our first hypothesis.

## Materials and Methods

### Participants and Datasets

**Figure 1:**
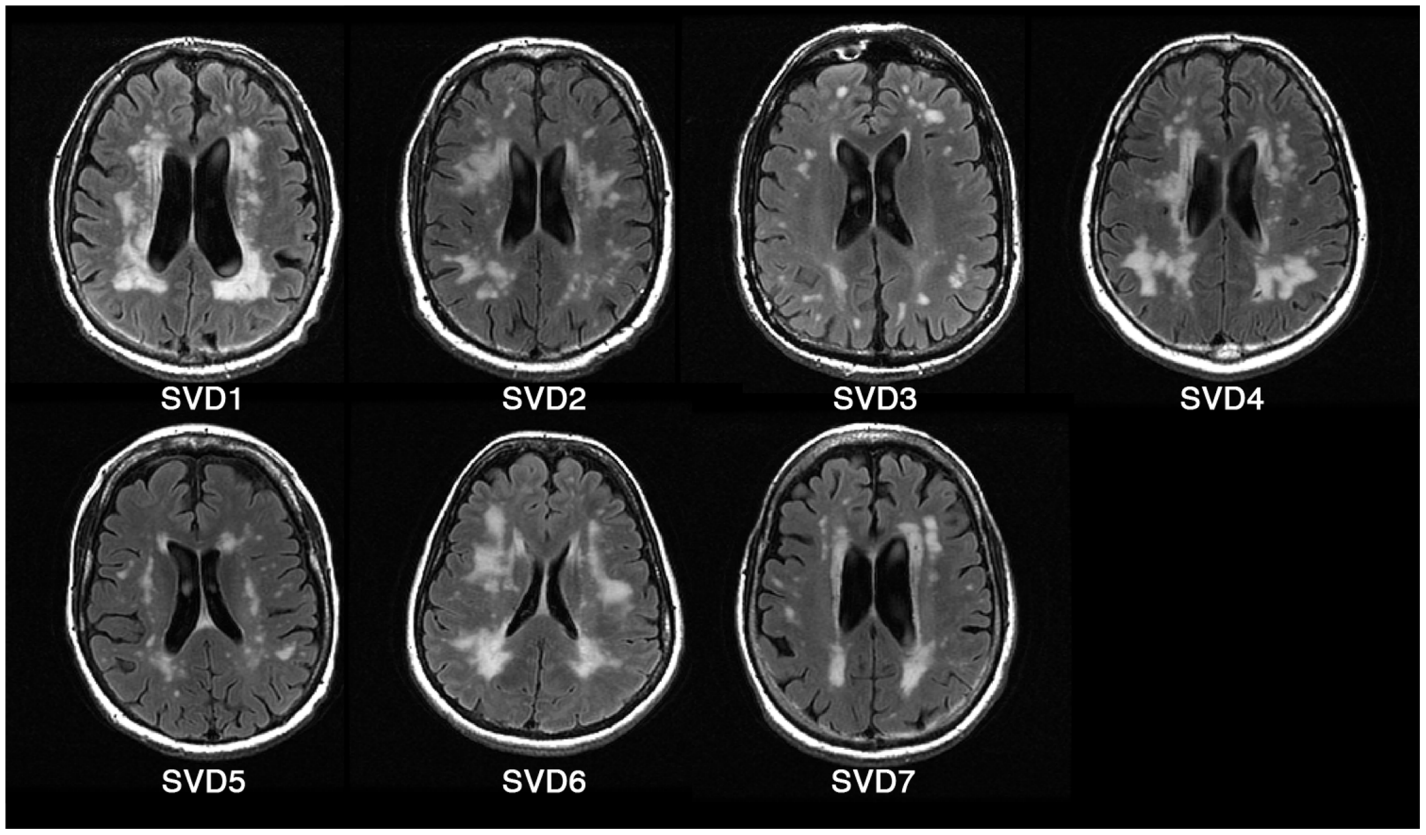
Fluid attenuated inversion recovery (FLAIR) images of SVD participants.

Two independent MRI datasets were included in this study. The primary dataset of twenty-eight participants was acquired in a cross-sectional study at Sunnybrook Health Sciences Centre and comprised three cohorts, referred to as the cardiac sampled BOLD data (cs-BOLD) (Table 1). Written informed consent was obtained from all participants and the Sunnybrook Research Ethics Board approved the study. Eleven participants were young healthy adult controls (YC), 10 were elderly healthy adult controls (EC), and 7 were elderly adults recruited from the cognitive neurology clinic with substantial cerebral small vessel disease (SVD). Figure 1 reflects the SVD cohort’s WMH burden. Exclusion criteria for all cohorts included psychiatric disorders, stroke, and dementia. Two of the SVD patients were diagnosed with cerebral autosomal dominant arteriopathy with subcortical infarcts and leukoencephalopathy (CADASIL), which can be considered a model of severe SVD [26]. The Montreal Cognitive Assessment (MOCA) score was 26±3.2 (standard deviation) for the SVD cohort and 27±1.2 for the EC cohort (one participant from this group did not have the MOCA administered due to time constraints). The YC cohort was drawn from graduate students and research assistants at Sunnybrook with an average of 17.4 years of education and was not screened using MOCA.

**Table 1:** Demographic information on the cs-BOLD cohorts.

Group	Young adult controls (YC)	Elderly adult controls (EC)	Small vessel disease (SVD)
No. of participants	11	10	7
Gender (M/F)	6/5	3/7	4/3
Age (mean ± SD) years	25.4±3.2	65.8±3.1	70±8.6

A second resting-state BOLD dataset, consisting of 84 participants from the International Consortium for Brain Mapping (ICBM) study was included in the current investigation. These data are referred to as the whole-brain BOLD or wb-BOLD cohort (19–85 years, mean = 44.3±17.9 years, 40 males, 44 females) and downloaded from http://www.nitrc.org/projects/fcon_1000/ [27].

### MRI Acquisition: cs-BOLD Data

**Figure 2:**
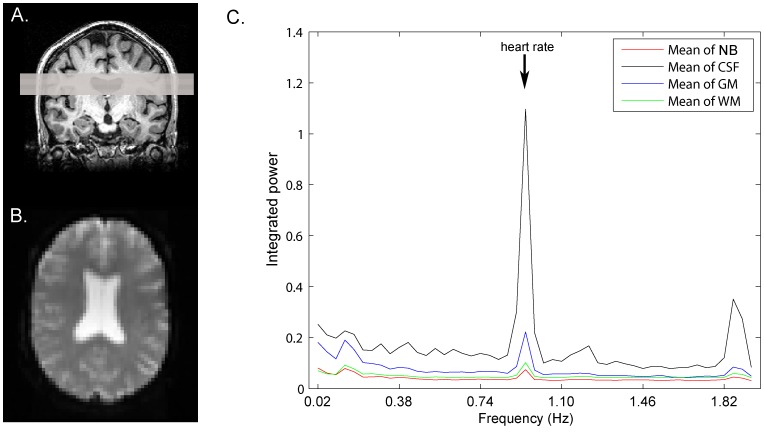
Overview of cs-BOLD imaging and the cardiac pulsatility metric. A) Axial positioning of the critically-sampled BOLD slices; B) Representative cs-BOLD axial slice from an elderly control at time point 1 (maximum tissue type contrast, non steady-state); C) smoothed mean tissue class power spectrum (each bin contains the integrated power within ±0.02 Hz of the central frequency). NB: non-brain voxels (pulsatility present because of EPI ghosting); CSF: cerebrospinal fluid; GM: grey matter; WM: white matter.

CS-BOLD data acquisition was performed using a GE 750 3 T scanner (Waukesha, WI). Resting-state images of each participant were acquired axially using an EPI sequence with the following parameters: flip angle = 34°, TR/TE = 250/30 ms, imaging matrix = 64×64, FOV = 192×192 mm. The resulting voxel resolution was 3×3×5 mm, and 4 slices were acquired for a total of 510 volumes over 2∶08 min. The imaging volume was positioned axially at the superior portion of the lateral ventricles (Figure 2 A, B) because this is an anatomical region highly susceptible to WMH formation. The superior slice was positioned outside the ventricles and the bottom slices cut across the ventricles. High resolution, whole brain anatomical T1 weighted images were also acquired with the following parameters: flip angle = 8°, TR = 8.2 ms, min/max TE = 3.2/13 ms, imaging matrix = 256×192, FOV = 22×16.5 mm, slice thickness = 1 mm. T2 weighted fluid attenuated inversion recovery (FLAIR) imaging was performed for WMH segmentation using the following parameters: TR = 9,700 ms, TE = 140 ms, imaging matrix = 256×192, FOV = 22 mm, slice thickness = 3 mm, TI = 2,200 ms. Whole brain EPI reference scans were acquired to facilitate coregistration of cs-BOLD slabs to anatomical T1 images (flip angle = 90°, TR = 3 seconds, TE = 30 ms, imaging matrix = 64×64, FOV = 192×192 mm, slice thickness = 5 mm, time points = 3). An InVivo Magnitude 3150 M MR compatible pulse oximeter (Gainesville, FL) was used to record the participants’ heart rates during resting state scans.

### MRI Acquisition: wb-BOLD Data

The EPI resting-state scans from this dataset were acquired on a 3 T scanner with a TR = 2 seconds, TE = 50 ms, 128 volumes, 23 axial slices, and a 4×4 mm in-plane resolution. Of the 84 total scans, 40 were acquired with a slice thickness of 5.5 mm and 44 with a slice thickness of 4 mm. Information concerning this dataset and project can be found at www.nitrc.org.

### MRI Data Processing

Both the wb-BOLD and cs-BOLD datasets were preprocessed using similar steps. The skull was removed from T1 images using the brain extraction tool (BET, Oxford Centre for Functional MRI of the Brain, Oxford, England) [28]. Resulting T1 images were segmented into white matter, grey matter, and cerebrospinal fluid using “FMRIB’s Automated Segmentation Tool” (FAST, Oxford Centre for Functional MRI of the Brain) [29]. FSL version 4.1.9 was used for all analyses. WMH were segmented on FLAIR using Fuzzy Lesion Extractor (FLEX) [30]. The EPI time series data were motion corrected using “Motion Correction using FMRIB’s Linear Image Registration Tool” (MCFLIRT, Oxford Centre for Functional MRI of the Brain) [31]. The first 10 volumes of each EPI series in the cs-BOLD dataset were discarded; no volumes were discarded from the wb-BOLD dataset because the first 5 volumes had already been discarded prior to the data’s release. Resting-state scans were then coregistered to T1 images, either directly, as for the wb-BOLD dataset, or by using the whole brain EPI dataset as an intermediate step, as for the cs-BOLD dataset. An initial standard registration (from EPI to T1) was performed for all participants using “FMRIB’s Linear Image Registration Tool” (FLIRT, Oxford Centre for Functional MRI of the Brain) [32]. If a visual inspection revealed obvious inferior-superior misalignment then a weighted local Pearson coefficient function created specifically for coregistration of BOLD fMRI to T1 was used (AFNI, NIMH, Bethesda, Maryland) [33]. If the secondary method also yielded obvious misalignment then registration was performed using an axial slab extracted from the T1 at the appropriate level. Unsatisfactory inferior-superior alignment occurred for some participants in the cs-BOLD dataset due to limited axial coverage of the cs-BOLD images (3 in the YC cohort, 0 in the EC, and 0 in the SVD). Unsatisfactory alignment was defined as a visible axial shift of more than half the EPI slice thickness. FLIRT yielded satisfactory alignment for all whole-brain scans from the wb-BOLD dataset.

Inverse matrices were used to transform tissue class masks from T1 space to EPI space and enable analysis in native subject space. WMH masks were coregistered to EPI space from FLAIR by concatenating the transformation matrices from T1 to EPI and from FLAIR to T1. After the transformation from T1 to EPI space, tissue class masks were eroded using a 3 mm full width at half maximum Gaussian kernel to reduce partial volume effects.

### Physiological Noise Metric for cs-BOLD and wb-BOLD Data

A physiological noise metric, σ_physio_, was calculated on a voxel-wise basis. The method described previously by Yacoub et al. [34] was used. Briefly, the square summation law, σ^2^_EPI_ = σ^2^_physio_+σ^2^_therm_, was employed to subtract the variance in signal attributed to thermal noise from the total variance. σ^2^_EPI_ is the total temporal variance at a particular voxel (GM, WM, or WMH), σ^2^_physio_ is the temporal variance at that voxel due to physiological processes (referred to as physiological noise), and σ_therm_^2^ is the temporal variance due to thermal noise. Temporal variances were calculated over the entire 500 point time course.
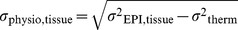
(1)where tissue = GM, WM or WMH.

Thermal noise was measured as the average variance in a region of interest (ROI) placed outside the brain in a corner of each resting-state image; this placement was chosen to minimize contribution from the EPI ghosting artifact. The σ_physio_ was normalized to each voxel’s mean signal intensity and expressed as a percentage; the average value was reported for each participant. Physiological noise was calculated by tissue type: GM, WM and WMH. In the case of wb-BOLD data, WM and WMH tissue types were merged since this dataset did not include FLAIR images.

### Cardiac Pulsatility Metric for cs-BOLD Data

To determine the relative BOLD signal change at the cardiac frequency, a voxelwise “cardiac pulsatility” metric was calculated. A fast Fourier transform was applied to each voxel’s time course to transform signal fluctuations to the frequency domain. Given a TR of 0.25 seconds, the Nyquist theorem states that signal fluctuations at up to 2 Hz could be detected without aliasing. The cardiac frequency was identified using pulse oximetry data and the spectral amplitude within 0.02 Hz of the cardiac frequency was integrated and divided by the frequency domain (0.04 Hz) to obtain average amplitude. This average amplitude was then normalized to each voxel’s mean intensity. A frequency range of ±0.02 Hz was chosen based on manual evaluation of the average width of the cardiac peak. The resulting metric was a voxel wise percentage signal change at the cardiac frequency and the average value was reported for each participant and tissue type. Cardiac pulsatility was not calculated for the wb-BOLD dataset because the sampling frequency of 0.5 Hz was not sufficient to isolate BOLD fluctuations at the heart rate. A mean tissue class frequency spectrum is shown in Figure 2C.

### Cumulative Head Motion as a Covariate of Non-interest: cs-BOLD Data

Head motion accumulated over the cs-BOLD scan was calculated to determine whether gross motion was 1) different between groups (unpaired t-test) or 2) correlated with physiological noise metrics (Pearson’s correlation). FLIRT’s root mean squared head displacement relative to previous TR was summed over the 500 cs-BOLD volumes.

### Statistical Analysis and Post-hoc Considerations

To address our main hypotheses, Analysis of Variance (ANOVA) tests were performed using SPSS Statistics 19 (IBM Inc., Chicago, Illinois). In all cases two tail p value statistics are reported and considered significant, unless stated otherwise, at p = 0.05. The following tests were performed: 1) WM cs-BOLD group differences on the σ_physio_ metric, 2) WM cs-BOLD group differences on the cardiac pulsatility metric, 3) a intra-subject comparison of physiological noise between NAWM and GM, 4) ANCOVA tests were the same as in 1 and 2 but with thermal noise, white matter volume and white matter partial volume estimate as covariates of non-interest, 5) a within-SVD cohort paired t-test for the differences between NAWM and WMH, with reported Cohen’s D effect sizes for the two metrics, 6) a group comparison of head motion during cs-BOLD. Test 4 was performed evaluate the potential effect of temporal (i.e. thermal noise) and spatial nuisance (white matter characteristics) factors that may influence the physiological noise metrics. Finally, a linear regression model was used on the ICBM data to support test 1 above and characterize an aging effect on the σ_physio_ metric.

## Results

### Aging and Disease Effects on Physiological Noise in WM and GM/WM Tissue Contrast: cs-BOLD Data

**Figure 3:**
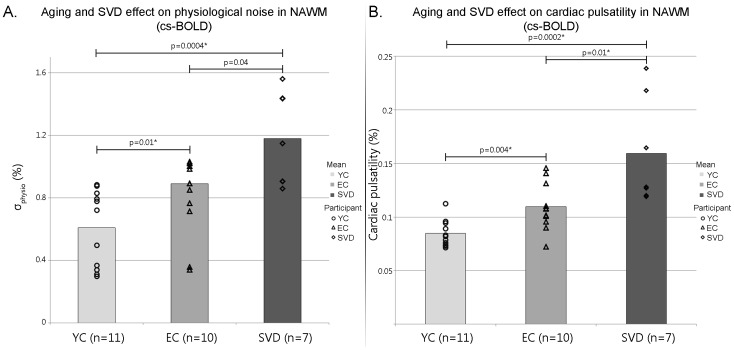
cs-BOLD physiological noise and cardiac pulsatility results. (A) Mean physiological noise (σ_physio_) in the NAWM of young controls (YC), elderly controls (EC), and patients with small vessel disease (SVD); (B) mean cardiac pulsatility in the NAWM of YC, EC, and SVD. *: significant after Bonferroni correction for multiple comparisons.

There was a statistically significant difference between σphysio in the YC, EC and SVD groups as determined by one-way ANOVA (F(2,25) = 13.5, p<0.0005). Results of post-hoc unpaired t-tests are provided in Figure 3 A. Physiological noise increased from YC to EC (p<0.01, t = 2.8), from EC to age matched SVD patients (p<0.04, t = 2.3), and from YC to SVD (p<0.0004, t = 4.4). After Bonferroni correction for multiple comparisons all results remained significant except for EC to SVD. Within participants, GM and WM regions were found to have different σphysio levels in 20/21 healthy controls and 5/7 SVD patients (Table 2). The trend towards diminished contrast between GM and NAWM in the SVD cohort is consistent with increased physiological noise in the NAWM of patients with SVD (Figure 3 A). The selection of regions outside the brain (i.e. 4 corners of the EPI data versus 1 corner) to estimate thermal noise did not influence the σphysio metric (i.e. less than a 1% difference on the estimated metric).

**Table 2:** The proportion of participants that showed NAWM was reduced compared to GM or equivalent to its level in terms of (A) σphysio or (B) cardiac pulsatility.

	Physiological noise			Cardiac pulsatility		
(Participants)/(cohort size)	YC	EC	SVD	YC	EC	SVD
NAWM<GM	10/11	10/10	5/7	10/11	10/10	2/7
NAWM = GM	1/11	0/10	2/7	1/11	0/10	5/7

The intra-subject tissue type comparison was significant if p<0.05.

### Evaluation of Normality and Homogeneity of Variance

Physiological noise had homogeneous variance across the YC, EC, and SVD cohorts for all tissue classes (Levene’s statistic of 0.67, 1.1, and 2.9 and p-values of 0.52, 0.36, and 0.072 for CSF, GM, and WM respectively). Normality was evaluated for all tissue classes using the Shapiro-Wilk test (p-values of 0.37, 0.85, and 0.11 for CSF; 0.12, 0.82, and 0.86 for GM; and 0.03, 0.35, and 0.13 for WM in the YC, EC, and SVD cohorts respectively).

### Aging and Disease Effects on Cardiac Pulsatility in WM and GM/WM Tissue Contrast: cs-BOLD Data

**Figure 4:**
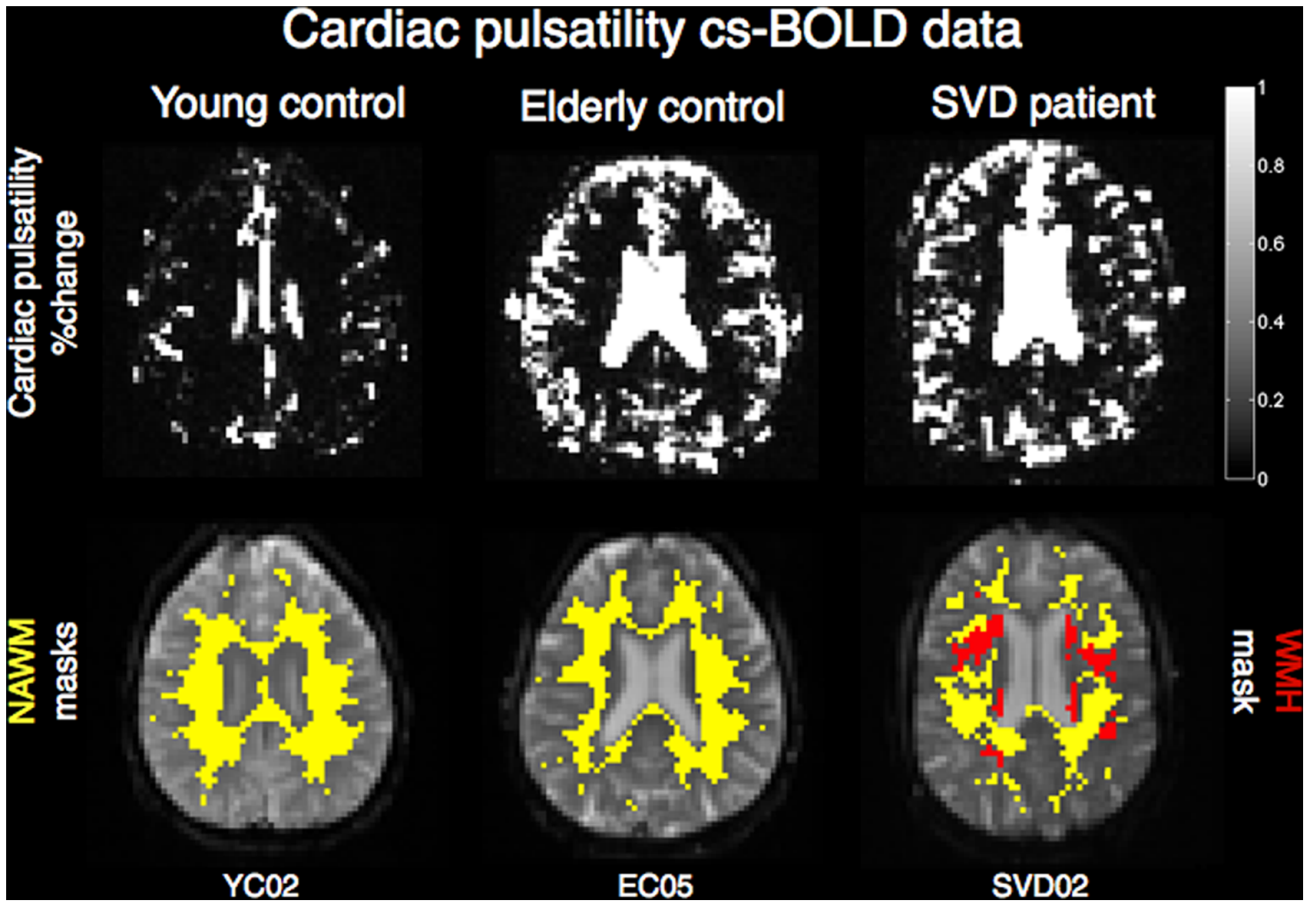
Illustrative cardiac pulsatility slices. Top: cardiac pulsatility in three cs-BOLD participants representative of the YC, EC, and SVD cohorts; bottom: NAWM masks (yellow) overlaid on cs-BOLD images for the same participants, WMH mask (red).

An illustration of the cardiac pulsatility images for three representative participants is shown in Figure 4. There was a statistically significant difference in WM cardiac pulsatility between YC, EC and SVD groups as determined by one-way ANOVA (F(2,25) = 14.0, p<0.0005). Results of post-hoc unpaired t-tests are provided in Figure 3 B. Cardiac pulsatility in NAWM increased with aging (p<0.004, t = 3.3 for comparison between YC and EC), the presence of SVD (p<0.01, t = 3.0 for comparison between EC and SVD), and from YC to SVD (p<0.0002, t = 4.8). All results remained significant after Bonferroni correction for multiple comparisons. Within participants, GM and WM regions were found to have different cardiac pulsatility levels in 20/21 healthy controls and 2/7 SVD patients (Table 2). The trend towards diminished contrast between GM and NAWM in the SVD cohort is consistent with increased cardiac pulsatility in the NAWM of patients with SVD (Figure 3 B).

### Effect of Thermal Noise, WM Volume & Partial Volume as Covariates: cs-BOLD Data

The cs-BOLD group differences in σ_physio_ and cardiac pulsatility remained significant when accounting thermal noise as a covariate (F(2,24) = 4.8, p<0.02 and F(2,24) = 8.8, p<0.001, respectively). After including WM volume and WM partial volume estimate as two additional covariates in the cs-BOLD data, the F statistic for σ_physio_ was not significant (F(2,22) = 1.8, p = 0.18), however cardiac pulsatility remained significant (F(2,22) = 4.6, p<0.02). This may be attributed to the fact that physiological noise was calculated over the entire power spectrum and therefore may be less specific to cardiac pulsation effects than cardiac pulsatility.

### Physiological Noise is Lower in WMH than in NAWM: cs-BOLD Data

**Figure 5:**
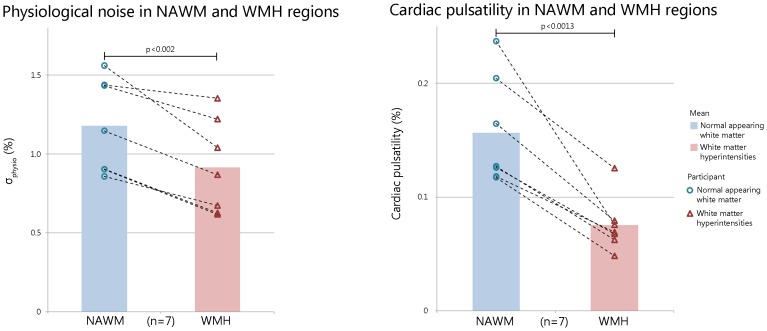
Comparison between white matter hyperintensities (WMH) and normal appearing white matter (NAWM) using (A) physiological noise and (B) cardiac pulsatility metrics.

Within the SVD cohort, cardiac pulsatility and σ_physio_ were reduced in WMH regions compared to NAWM regions (paired t-tests, p<0.0013, t = 5.7; and p<0.002, t = 5.2, respectively, Figure 5). The sensitivity to distinguish WMH lesions from NAWM was higher when using the cardiac pulsatility metric compared to the physiological noise metric (effect sizes = 2.14 and 0.88 respectively). The WMH volume was significantly correlated with σ_physio_ but not cardiac pulsatility (p<0.03 and p = 0.37 respectively). NAWM volume was not correlated with either metric (p = 0.19 and p = 0.57).

### Head Motion During cs-BOLD

Cumulative head motion over 500 volumes was not significantly different between EC and SVD cohorts (23 and 29 mm respectively, p = 0.16, t = 1.46). Cumulative head motion was also not significantly correlated to physiological noise in either the EC or SVD cohort (Pearson correlation coefficients of 0.34 and 0.14 respectively, p-values of 0.34 and 0.76 for the same cohorts). Head motion was not correlated to age in any of the cs-BOLD cohorts (Pearson correlation coefficients of 0.51, 0.03, and 0.31 for the YC, EC, and SVD cohorts respectively; p-values of 0.15, 0.94, and 0.61 for the same cohorts).

### Aging Effect on Physiological Noise in WM: wb-BOLD Data

**Figure 6:**
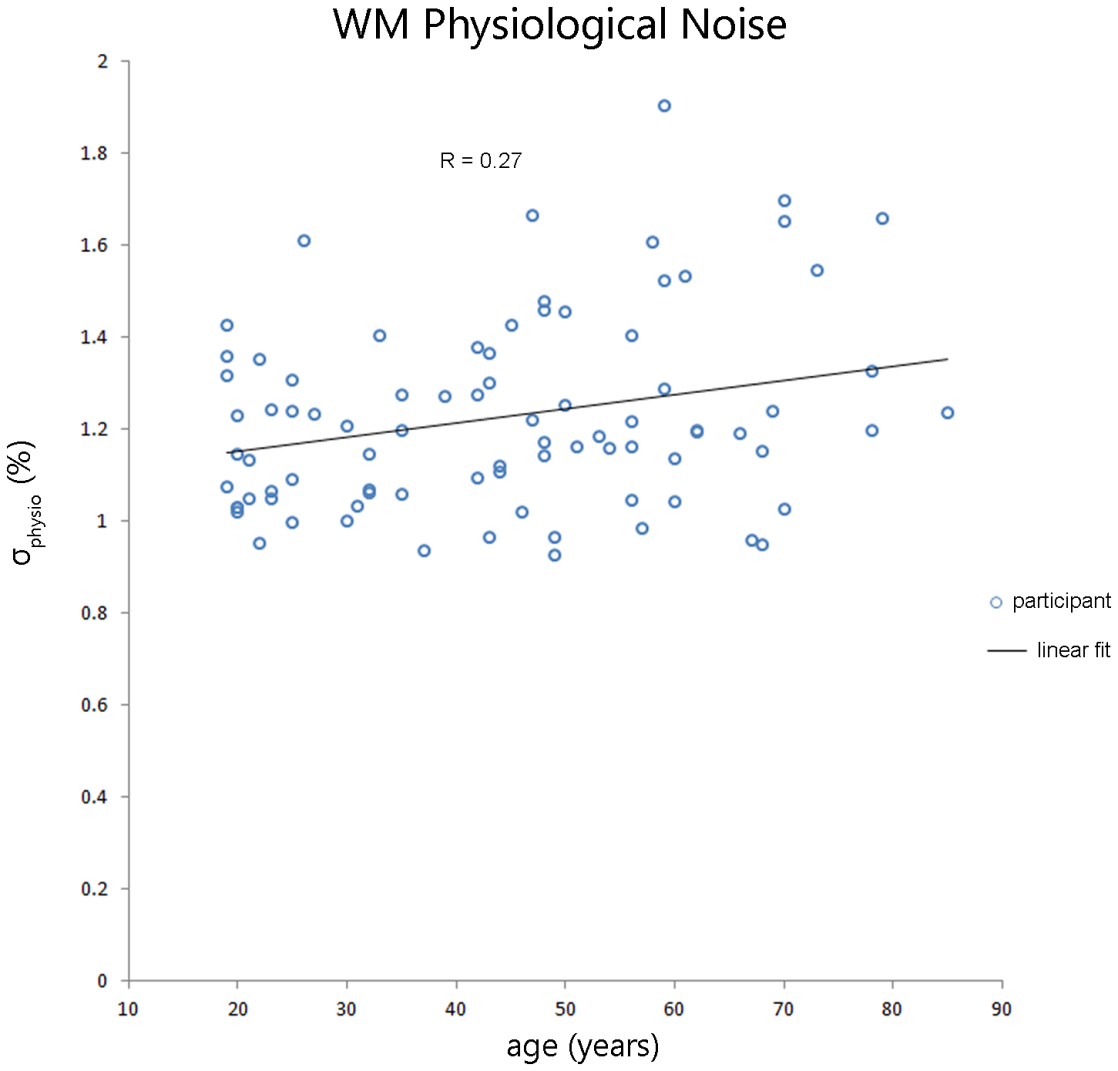
Significant age effect on physiological noise in WM in wb-BOLD data (TR = 2s, n = 84, r = 0.27, p<0.01).

Figure 6 shows σ_physio_ increased with age in WM for the wb-BOLD (TR = 2s). A linear regression showed a significant age effect (p<0.01, r = 0.27) and remained significant when slice thickness was included in the model (p<0.007). Residuals were assessed using a plot of residuals versus predicted values and a quantile-quantile (QQ) plot. The residuals were randomly distributed. Two wb-BOLD sub-groups were chosen to match the cs-BOLD data (i.e. 32 young adults, 20≤age≤35 years, mean age = 25.3 and 27 older adults, 55≤age≤85 years, mean age = 65.3 years). We found a significant age-related WM σ_physio_ difference (F(1,57) = 5.8, p<0.02), which remained significant with thermal noise as a covariate (F(1,56) = 5.7, p<0.021). A tissue type contrast between GM and WM was performed and all 84/84 participants had a significantly greater σ_physio_ in GM than in WM (p<0.05 for each intra-subject comparison, on average 31% greater σ_physio_ in GM).

### No Sex Effect on Physiological Noise in WM: wb-BOLD and cs-BOLD Data

The effect of sex on physiological noise was not significant in the larger wb-BOLD dataset (mean σ_physio_ WM of 0.99 and 0.94 for males and females respectively, p = 0.1 and t = 1.65). In the smaller cs-BOLD dataset, the effect of sex on physiological noise was not significant in any of the cohorts. The mean σ_physio_ WM for males and females respectively were 0.60 and 0.63 in the YC cohort (p = 0.84, t = 0.22); 1.1 and 0.9 in the EC cohort (p = 0.12, t = 1.7); and 1.3 and 0.97 in the SVD cohort (p = 0.11, t = 1.9).

## Discussion

This study found that whole brain cardiac sampled BOLD (TR = 0.25 s) could be used to identify aging and small vessel disease pathology effects within white matter. Physiological noise in cs-BOLD data increased with age in the NAWM of healthy adults, an effect that could not be explained by thermal noise and was corroborated by a parallel analysis performed on publically available whole brain BOLD data (TR = 2 s). Significant cs-BOLD findings were also observed when considering the older participants. First, physiological noise was increased in NAWM in adults with SVD compared to age-matched controls. Second, within the SVD cohort itself, physiological noise was decreased in the regions defined by the white matter hyperintensities (WMH) when compared to NAWM.

A FLAIR MR image provides excellent contrast to visualize WMH as manifestations of SVD. In this study we provide evidence that global WM metrics of dynamic and/or pulsatile information can also be used to differentiate WM tissue types and groups. These approaches may help to advance our understanding SVD given that others argue that static MR imaging approaches may be inadequate [35]. In the current study, the NAWM physiological noise and cardiac pulsatility metrics were higher in SVD patients compared to age-matched elderly controls (Figure 3), which is consistent with the notion that SVD contributes to advanced aging and that this can be seen in NAWM [35]. For instance, O’Sullivan et al. used diffusion-tensor imaging to show that mean diffusivity increased and FA is reduced in regions of NAWM of SVD patients compared to controls [36]. From hemodynamic based measures, others provide evidence for global hemodynamic effects in relation to SVD. Cerebral arterial stiffness assessed by the transcranial Doppler ultrasound pulsatility index was correlated with the WMH lesion volume [37] and more recently with the Fazekas scale [23]. Others have used histology in patients with SVD to identify blood vessel abnormalities [38] and an increased expression of major histocompatibility complex II from microglia in the NAWM from individuals with SVD [39].

Regions where WMH were present showed the opposite trend compared to the NAWM aging observation, in the sense that physiological noise and cardiac pulsatility were decreased in WMH compared to NAWM. Reduced cardiac pulsatility in the WMH lesion itself may be explained in part by the following. First, Brown et al. found a decreased concentration of afferent vessels within WMH regions compared to NAWM on histological analysis [40]; a reduced vascular density could contribute to decreased pulsatility. A second example comes from an overt stroke study whereby the ischemic lesion had reduced physiological noise compared to intact tissue [41]. A final perspective on white matter cardiac pulsatility in SVD pertains to the venous system. Moody and colleagues proposed that SVD is associated with thickening of vein walls due to the deposition of collagen that could decrease any cardiac pulsatility effects on the venous side [42]–[43]. A recent MRI and histology autopsy study in AD brains found that collagenosis in large veins (i.e. 200 micron size) significantly predicted the WMH lesion volume [44]. Thus, upstream or downstream vessels may contribute to the cardiac pulsatility in the small vessels in white matter. Additionally, physiological noise in NAWM was positively associated with the volume of the lesion burden, which suggests that global cerebrovascular measures may be useful in characterizing WMH pathology.

This study has limitations that are addressed by the following. First, the wb-BOLD data served as a means to validate the σ_physio_ findings from the cs-BOLD data but the two datasets had some discrepant imaging parameters. In addition to the TR difference, 2 s for whole brain versus 0.25 s for unaliased cardiac sampling, TE, slice thickness and voxel dimensions were different, which could influence σ_physio_. Second, the cs-BOLD data had a relatively small SVD cohort that consisted of adults with extensive SVD, of which two were individuals with a CADASIL diagnosis. In addition the SVD and EC groups were not well matched in age and sex. Another issue was the lack of FLAIR images from the wb-BOLD dataset that would be used to characterize the WM as NAWM or WMH, as was performed in the cs-BOLD data. Finally, atrophy in WM is a consequence of aging [45]. WM volume and WM partial volume estimates as covariates did render the age effect on σ_physio_ as non-significant, however, the cardiac pulsatility aging effect did remain significant (p<0.021) and provides some support for the use of the cardiac pulsatility as a sensitive physiological metric.

The cs-BOLD sequence used a short TR (0.25 s) to sample unaliased cardiac signals at the expense of brain coverage in the z-direction (20 mm). We established the proof of principle that global WM indices of physiological noise can be used, however, future work could involve voxel-wise analyses to identify specific locations of heightened cardiac pulsatility effects. Other potentially useful areas of future work could include characterization of inflow effects [46] using diffusion encoding gradients, saturation pulses [47] or multi-echo EPI [48] strategies to address the inflow contribution to the cs-BOLD contrast. Another approach would be to compare stiffness between NAWM and WMH using MR elastography [49]. Finally, using parallel imaging technologies, it is possible to perform cs-BOLD with whole brain coverage while still maintaining a high temporal resolution [50]. Rapidly sampled BOLD data may have wider appeal in characterization of the temporal frequency features in resting state fMRI in both grey and white matter.

### Conclusion

The current study made use of a stock BOLD fMRI pulse sequence with reduced TR (0.25 s) and a short scan duration (2∶08 m) to demonstrate increased physiological noise and cardiac pulsatility in NAWM in aging and additionally the presence of SVD. The WMH lesion volume itself had reduced physiological noise compared to NAWM, while the physiological noise in NAWM of adults with SVD was higher compared to NAWM in elderly controls. White matter cardiac frequency physiological signal in resting state BOLD data may reflect the increased cerebral blood volume fluctuations that are due to pulsatile energy that is transmitted down the vascular tree to the small vessels. This relatively straightforward technique could prove useful in the clinical characterization of SVD abnormalities that are not readily identified with conventional static MR imaging.
